# Vitamin D Deficiency in Autism Spectrum Disorder: A Cross-Sectional Study

**DOI:** 10.1155/2020/9292560

**Published:** 2020-09-18

**Authors:** Maria G. Petruzzelli, Lucia Marzulli, Francesco Margari, Andrea De Giacomo, Alessandra Gabellone, Orazio V. Giannico, Lucia Margari

**Affiliations:** ^1^Department of Basic Medical Sciences, Neuroscience and Sense Organs, University of Bari “Aldo Moro”, Bari, Italy; ^2^Department of Biomedical Sciences and Human Oncology, University of Bari “Aldo Moro”, Bari, Italy

## Abstract

Vitamin D plays a role in central nervous system (CNS) development. Recent literature focused on vitamin D status in children and adolescents with autism spectrum disorder (ASD), but with inconsistent results. Our case-control study is aimed at evaluating serum 25-hydroxyl-vitamin D (25(OH)D) concentration in children with ASD (ASD group, *n* = 54) compared to children affected by other neurological and psychiatric disorders (non-ASD group, *n* = 36). All patients were admitted at the Complex Operative Unit of Child Neuropsychiatry, Polyclinic of Bari, Italy. 25(OH)D was quantified by chemiluminescence immunoassay and level defined as: deficiency (<20 ng/mL); insufficiency (20–30); normality (30-100); toxicity (>100). Statistical analysis was performed using SPSS20 (significance < 0.05). The ASD group showed 25(OH)D a mean level significantly lower than control (*p* = 0.014). Multivariable logistic regression analysis showed an association between ASD and vitamin D deficiency (*p* = 0.006). The nature of such association is unclear. Vitamin D deficiency may probably act as a risk factor for the development of ASD. Further studies are needed to unravel the role of vitamin D in ASD etiology and investigate its therapeutic potential.

## 1. Introduction

Vitamin D is a member of the family of steroid hormones mainly existing in two forms, ergocalciferol (vitamin D2), which is photochemically synthesized by plants, and cholecalciferol (vitamin D3), which is synthesized in the skin in the presence of ultraviolet rays B (UVB) resulting from sun exposure. In humans, a sequential 25-hydroxylation and 1*α*-hydroxylation of vitamin D2 and vitamin D3 precursors take place in the liver and in the kidney, respectively, resulting in the synthesis of 25-hydroxycholecalciferol (25(OH)D) in the former and 1,25 dihydroxycholecalciferol (1,25(OH)2D) in the latter [1, 2]. The active metabolite 1,25(OH)2-D, also called calcitriol, plays a direct action on bone tissue cells, activating osteoclastogenesis and bone resorption, and an indirect action, through the regulation of phosphocalcium homeostasis. In addition, the presence of vitamin D receptors (VDR) and enzymes involved in Vitamin D metabolism in tissues other than bone suggests that calcitriol carries out many other functions, including the regulation of innate and adaptive immunity and plays a neurotrophic and antioxidant role in the central nervous system (CNS) [3]. According to the Endocrine Society's Practice Guidelines, vitamin D deficiency is defined as 25(OH)D serum concentration < 20 ng/mL, insufficiency as 21–29 ng/mL and sufficiency as at least 30 ng/mL for maximum musculoskeletal health [4]. It has been estimated that approximately 30% and 60% of children and adults worldwide are vitamin D deficient and insufficient, respectively [4]. In recent years, the extraskeletal impact of vitamin D deficiency has been an active area of research [2]. Epidemiological and case-control studies have often suggested a link between vitamin D deficiency and conditions such as type 1 and type 2 diabetes, connective tissue disorders, inflammatory bowel disorders, chronic hepatitis, food allergies, asthma and respiratory infections, and cancer [2]. The relationship between vitamin D deficiency and neurodevelopmental disorders is less clear, but, with the introduction of vitamin D into the broader family of neuroactive steroids [3], it is now conceivable that optimal levels of vitamin D are also necessary to preserve the neurological development and to protect the brain [1]. Autism spectrum disorder (ASD) is a part of a broad spectrum of neurodevelopmental disorders characterized by impairments of social interaction, verbal and nonverbal communication, and behaviour. ASD etiology is still unknown, but both genetic and environmental factors are hypothesized to play a causative role. Several observational studies have examined the association between vitamin D status and ASD in children and adolescents, achieving inconsistent results. Many case-control studies revealed that children with ASD have lower vitamin D concentrations than their healthy counterpart, suggesting that D-hypovitaminosis may be a risk factor for ASD (3, vb, vb), and vitamin D supplementation useful for treating its symptoms. Here, we sought to produce further insight in this field by comparing serum levels of vitamin D between children with ASD and a control group of non-ASD patients.

## 2. Materials and Methods

### 2.1. Participants

A retrospective chart review of records from inpatients referred to Child Neuropsychiatry Unit of University of Bari “Aldo Moro” from 2014 to 2018 was performed. According to the aim of the study, we selected all subjects responding to the following inclusion criteria: patients of both sex, under the age of 18, whose 25(OH)D level was available. The missing data on 25(OH)-vitamin D level or the evidence, from medical history, physical examination, and laboratory findings, of severe malnutrition or chronic diseases, potentially affecting normal vitamin D metabolism, were considered as exclusion criteria.

All the enrolled patients were subdivided into two groups, respectively, including children identified with ASD and children with other neuropsychiatric disorders different from ASD.

ASD diagnoses were defined by experienced child and adolescent psychiatrists according to the Diagnostic and Statistical Manual of mental disorders (DSM-5) criteria [5]. The diagnostic process, based on child observation and interaction, as well as on clinical interview with parents, was supported by administration of Autism Diagnostic Interview-Revised (ADI-R) [6] and Autism Diagnostic Observation Schedule, Second Edition (ADOS-2) [7] by properly trained psychologists.

The ADI-R is a standardized, semistructured interview during which parents or carers report information about an individual suspected of having an ASD. It assesses behavior across three domains: reciprocal social interaction, communication and language, restricted and repetitive, and stereotyped interests and behaviors. The ADOS-2 is a semistructured assessment of communication, social interaction, play, and restricted and repetitive behaviors, widely accepted as a “gold standard” instrument for the diagnosis of ASD across age, developmental level, and language skills. One of four different modules may be administered according to the expressive language level and chronological age of the patient. All the evaluations were discussed in regular reliability meetings, supervised by the senior researcher.

### 2.2. Procedure

All patients underwent a comprehensive clinical assessment, including anamnestic interview, physical and neurological examination, and laboratory blood tests and instrumental evaluation by electrocardiogram, electroencephalogram, and brain magnetic resonance, when indicated. Serum concentrations of 25(OH)-D (the hepatic metabolite of vitamin D) were determined by chemiluminescence immunoassay and expressed as ng/ml. Ranges indicative of a state of deficiency, insufficiency, normality, or toxicity were determined as previously reported [4]:

(1) $$\begin{array}{c} \text{Deficiency}=25\left(\text{OH}\right)\text{D}<20\text{ ng}/\text{ml}\\ \text{Insufficiency}=20\text{ ng}/\text{ml}<25\left(\text{OH}\right)\text{D}<30\text{ ng}/\text{ml}\\ \text{Normality}=30\text{ ng}/\text{ml}<25\left(\text{OH}\right)\text{D}<100\text{ ng}/\text{ml}\\ \text{Toxicity}=25\left(\text{OH}\right)\text{D}>100\text{ ng}/\text{ml} \end{array}$$

Written informed consents were achieved from parents, and the study was approved by the Ethical Committee of the Hospital Consortium Policlinic of Bari.

### 2.3. Statistical Analysis

Statistical analysis was performed using SPSS 20. Statistical significance *α* was fixed to 0.05. Categorical variables were reported as absolute and relative frequencies. Continuous variables were reported as median and IQR and compared between ASD and non-ASD patients through the Wilcoxon rank sum test in order to account for nonnormality, evaluated through the Shapiro-Wilk test, and heteroscedasticity, evaluated through the Bartlett test. The minimum sample size calculated through Altman's monogram was 30 for each group with a statistical power of 80% and a standardized difference of 1.

A multivariable binary logistic regression model for vitamin D, age, and sex was then fitted in order to assess the effect of vitamin D levels on the presence of ASD through the estimation of adjusted odds ratios. The sample size was idoneous relying on the one in ten rule. The goodness of fit of the model was evaluated through the Hosmer Lemeshow test and the pseudo *R*^2^ of Nagelkerke.

## 3. Results

The ASD group included 54 patients, while 36 subjects bearing other neuropsychiatric disorders were included in the control group. Demographic and clinical features of the two study groups are summarized in Table 1. The non-ASD group included 15 patients with diagnosis of neurodevelopmental disorders other than ASD (4 with intellectual disability, 3 with attention-deficit/hyperactivity disorder, 3 with motor disorders, 2 with specific learning disorder, and 3 with language disorder), 11 patients with diagnosis of psychiatric disorder (5 with depressive disorders, 3 with an anxiety disorders, 2 with obsessive-compulsive disorder, and 1 with psychotic disorder), and 10 patients with neurological diseases (5 with epilepsy, 3 with migraine, and 2 with a peripheral neuropathy).

A multivariable logistic regression analysis showed that ASD was significantly associated (*p* = 0.006) with lower levels of vitamin D (Table 3). Moreover, the odds of 25(OH)D deficiency in ASD subjects appeared 10.31 times higher than those in the control group.

As reported in Table 2, the mean level of serum 25(OH)D was significantly lower in the ASD group as compared to non-ASD group (18.61 ± 8.33 ng/mL versus 24.62 ± 13.18 ng/mL; *p* = 0.014).

## 4. Discussion

The main finding of this study consisted in a significant association between ASD and the occurrence of Vitamin D deficiency (25(OH)D < 20 ng/ml). Such result appears of interest if one takes into account that a remarkable prevalence of vitamin D deficiency is emerging in the general population worldwide [4]. Recent epidemiological data in Italy reported a prevalence of vitamin D deficiency in line with European data, ranging 4–7%, 1–8%, and 12–40% according to the age intervals of 1–6 years, 7–14 years, and 15–18 years, respectively [8]. Consistent with the high prevalence of hypovitaminosis D into Italian pediatric population [8, 9], we found 25(OH)D mean levels < 30 ng/dL in both our clinical groups but, interestingly, ASD patients showed concentrations significantly lower than subjects with different neurological or psychiatric disorders. In a recent review [10], Wang et al. summarized results from 11 case-control studies comparing serum concentration of vitamin D between ASD children and healthy controls [11–21], supporting the hypothesis of an intriguing association between these two conditions [12, 14–18, 20, 21].

In the general population, D-hypovitaminosis is associated by limited sunlight exposure, low UVR level, low dietary vitamin D intake, non-Caucasian ethnicity, malabsorption syndromes, obesity, hepatic/renal failure, and some medications [4, 8]. All subjects included in this study are resident in the South of Italy, suggesting they are exposed to similar weather conditions. However, we are unable to exclude that specific lifestyle factors, as indoor environment, extensive clothing cover, or excessive sun avoidance may have led to different sunlight exposure between study patients. Similarly, repetitive and restricted dietary behaviors and food selectivity typically associated to ASD could negatively impact vitamin D dietary intake. A literature review [22] reported lower vitamin D dietary intakes in children with ASD compared to healthy controls [23–26], although this difference was not always statistically significant [27, 28].

Other authors hypothesize that D-hypovitaminosis is implicated in the ASD pathogenesis based on the severe vitamin D deficiency observed in this clinical setting, and our multivariable analysis is in line with such a picture. Modabbernia et al., in a sistematic evidence-based review, listed vitamin D deficiency as a nutritional risk factor for ASD [29]. It is currently thought that vitamin D deficiency cooperates with a wide range of additional environmental factors (obstetric complications, dysregulated steroidogenesis, maternal stress, maternal infection, etc.) in modulating multiple biological processes relevant to ASD, including calcium signaling abnormalities, mythocondrial dysfunctions, and neurotransmission [30]. Moreover, it is increasingly contemplated that neuroinflammation and immune dysfunctions play an etiologic role in different brain disorders, including autism, and, intriguingly, vitamin D is known to exert both anti-inflammatory and immunomodulatory effects in humans [31]. Over the last few years, a large body of research has been aimed at searching for links between ASD and aberrant immune function [32]. For instances, familial autoimmunity, maternal autoantibodies, and inflammation during gestation significantly increase the risk of ASD. Consistently, individuals with ASD show higher titers of autoantibodies and rates of immune dysfunctions or immune-mediated comorbidities than healthy population, and processes of neuroinflammation have been substantiated both *ex vivo* and in *post mortem* brain samples [33]. Two additional observations link vitamin D to immunity, the first standing in the capacity of immune cells to secrete vitamin D in both autocrine and paracrine fashion, the second based on the vitamin-D receptor (VDR) location in the brain as well as onto the surface of many immune cytotypes, as T- and B-cells, monocytes, macrophages, dendritic cells and neutrophilis [31]. Moreover, the VDR belongs to the neuro-steroid receptor family, thus further prompting future studies at investigating its role in ASD. Some reports showed the association of single nucleotide polymorphisms of the VDR gene with ASD, but their functional role is still far from being clarified. Finally, genetic aberrations of the VDR gene could affect vitamin D uptake and metabolism [34, 35], suggesting intriguing implications in the genetic susceptibility to ASD.

One of the major limitations of this study stands in its cross-sectional nature and small sample size. Furthermore, the absence of comparison with an additional healthy control sample and the assessment of influencing factors, as sun exposure and dietary patterns, should be considered as actual limitations and future direction of research.

## 5. Conclusion

Vitamin D deficiency due to genetic and/or environmental factors is common in ASD children and may represent a risk factor for developing ASD. Therefore, we believe that more frequent measurements of vitamin D levels in ASD patients may be considered a good clinical practice. Future prospective studies, supported by more translational experimental frameworks, should be aimed at clarifying the role of vitamin D in autism, even defining potential clinical benefits from its supplementation.

## Figures and Tables

**Table 1:** Demographic and clinical feature of the study samples.

	ASD (*n* = 54)	Non-ASD (*n* = 36)
Age (years) mean (±S.D.)	6.87 (±3.92)	11.28 (±4.44)
Gender		
Male *n* (%)	44 (81.5)	21 (58.3)
Female *n* (%)	10 (18.5)	15 (41.7)
Ethnicity	Caucasian	Caucasian
Residence	South of Italy	South of Italy

**Table 2:** Mann-Whitney U test for vitamin D levels distribution between ASD and non-ASD patients.

	Vit. D
Mann–Whitney *U*	673.500
Wilcoxon *W*	2158.500
*Z*	-2.460
Asymp. Sig. (2-tailed)	0.014

**Table 3:** Age- and sex-adjusted association between levels of vitamin D and ASD.

Vitamin D	ASD *n* (%)	Non-ASD *n* (%)	OR	95% CI for OR	*p* value
Normality	6 (11.1)	9 (25)	1.00	—	—
Insufficiency	13 (24.1)	15 (41.7)	3.29	0.61-17.71	0.166
Deficiency	35 (64.8)	12 (33.3)	10.31	1.96-54.22	**0.006**

## Data Availability

The data used to support the findings of this study are included within the supplemental information file.

## Supplementary Materials

Supplementary Materials Demographic features and vitamin D level for each ASD and non-ASD patient.
